# Usefulness of the Modified Videofluoroscopic Dysphagia Scale in Choosing the Feeding Method for Stroke Patients with Dysphagia

**DOI:** 10.3390/healthcare9060632

**Published:** 2021-05-27

**Authors:** Byung Joo Lee, Hyoshin Eo, Changbae Lee, Donghwi Park

**Affiliations:** 1 Department of Rehabilitation Medicine, Daegu Fatima Hospital, Daegu 44033, Korea; bjl84@naver.com (B.J.L.); wowo0011@naver.com (H.E.); 2 Department of Physical Medicine and Rehabilitation, College of Medicine, Ulsan University Hospital, University of Ulsan, Ulsan 44610, Korea; pclucky7@gmail.com

**Keywords:** deglutition, swallowing difficulty, dysphagia, videofluoroscopic dysphagia scale, videofluoroscopic swallowing study, modified version of the videofluoroscopic dysphagia scale

## Abstract

Introduction: The Videofluoroscopic Dysphagia Scale (VDS) is used to predict the long-term prognosis of dysphagia in patients with strokes. However, the inter-rater reliability of the VDS was low in a previous study. To overcome the mentioned limitations of the VDS, the modified version of the VDS (mVDS) was created and clinically applied to evaluate its usefulness in choosing the feeding method for stroke patients with dysphagia. Methods: The videofluoroscopic swallowing study (VFSS) data of 56 stroke patients with dysphagia were collected retrospectively. We investigated the presence of aspiration pneumonia and the selected feeding method. We also evaluated the correlations between the mVDS and the selected feeding method, and between the mVDS and the presence of aspiration pneumonia after stroke. Univariate logistic regression and receiver operating characteristic analyses were used in the data analysis. Results: The inter-rater reliability (Cronbach α value) of the total score of the mVDS was 0.886, which was consistent with very good inter-rater reliability. In all patients with dysphagia, the supratentorial stroke subgroup, and the infratentorial stroke subgroup, the mVDS scores were statistically correlated with the feeding method selected (*p* < 0.05) and the presence of aspiration pneumonia (*p* < 0.05). Conclusions: The mVDS can be a useful scale for quantifying the severity of dysphagia, and it can be a useful tool in the clinical setting and in studies for interpreting the VFSS findings in stroke patients with dysphagia. Further studies with a greater number of patients and various stroke etiologies are required for more generalized applications of the mVDS.

## 1. Introduction

Dysphagia is a serious clinical problem that can decrease the quality of life and lead to lethal conditions, such as aspiration pneumonia [1,2,3,4,5,6]. Although few clinical bedside tests are used universally, the videofluoroscopic swallowing study (VFSS) has been commonly accepted as the gold standard in assessing dysphagia [3,7,8]. The VFSS can detect aspiration and penetration in addition to various abnormalities in the oral, pharyngeal, and esophageal phases [9]. Therefore, it provides some guidance in determining which swallowing therapy should be applied and what type of diet should be adequate.

Among numerous methods to predict and quantify the prognosis of dysphagia, the Functional Dysphagia Scale is a useful tool that correlates well with the American Speech-Language-Hearing Association National Outcomes Measurement System [10,11]. However, despite its value in interpreting the severity of dysphagia, it does not predict the long-term prognosis, which is important because of the close relationship between prolonged dysphagia, high mortality, and a low respiratory tract infection rate [12].

The Videofluoroscopic Dysphagia Scale (VDS) is used to predict the long-term prognosis of dysphagia in patients with stroke [12,13]. Han et al. used the VDS to assess the long-term prognosis of dysphagia based on the development of any aspiration or penetration episode after 6 months from the onset of dysphagia [14]. The VDS consists of 14 categories and shows good correlation with an aspiration or a penetration symptom that occurs 6 months after the initial onset of dysphagia. [14]. The 14 items of the VDS represent oral functions (lip closure, mastication, bolus formation, premature bolus loss, apraxia, and oral transit time) and pharyngeal functions (pharyngeal triggering, laryngeal elevation and epiglottic closure, pharyngeal transit time, pharyngeal coating, vallecular and pyriform sinus residues, and aspiration) that can be observed from the VFSS video [14].

The VDS can also quantify the severity of dysphagia in total scores, but limitations regarding the subjectivity of the results have been noted in previous studies. Kim et al. reported the inter-rater reliability results of the VDS among 10 physiatrists [15]. In their study, the inter-rater reliability of the VDS showed a low rate of agreement (κ < 0.20), especially in bolus formation (κ = 0.153), mastication (κ = 0.123), apraxia (κ = 0.099), tongue to palate contact (κ = 0.153), premature bolus loss (κ = 0.060), and pharyngeal transit time (κ = 0.165) [15]. Other parameters showed a fair rate of agreement (κ > 0.2, κ < 0.4) [15]. Such results suggest that the VDS can be subjective according to interpreters, especially for several parameters, such as apraxia, tongue to palate contact, premature bolus loss, or bolus formation. Subjectivity is also possible because of the ambiguous criterion of several parameters of the VDS.

Therefore, to overcome the mentioned limitations of the VDS, some parameters of the VDS were modified in the present study. Furthermore, the modified version of the VDS (mVDS) was clinically applied to evaluate its usefulness in choosing the feeding method for stroke patients with dysphagia.

## 2. Methods

### 2.1. Ethics Statements

The protocol for this study was approved by the Institutional Review Board of Ulsan University Hospital (2021-01-028). This study was conducted according to the Declaration of Helsinki for human experiments. Informed consent was waived due to the retrospective nature of this study.

### 2.2. Study Design and Population

Data of stroke patients with dysphagia, who underwent a VFSS for the first time at Daegu Fatima Hospital between April 2019 and January 2021, were collected retrospectively. Patients who had any symptoms of difficulty in swallowing were recruited. We obtained clinical data such as sex, age, stroke onset, the Mini-Mental State Examination score, modified Bethel index score, type of lesions of stroke (surpatentorial or infratentorial lesions), history of aspiration pneumonia, and duration from stroke onset [16].

The criteria for inclusion were as follows: (1) a history of aspiration symptoms, such as coughing or choking; (2) symptoms clinically indicative of dysphagia, such as reduced gag reflex or delayed swallowing reflex; and (3) a history of using alternative feeding methods, such as a nasogastric tube. Patients who could not sit or those who had difficulty maintaining consciousness were excluded.

### 2.3. Aspiration Pneumonia

A retrospective review was conducted to investigate the development of aspiration pneumonia within 1 month before and after a VFSS in stroke patients with dysphagia [17,18]. The following data were collected: symptoms, such as coughing during feeding; the presence of sputum, dyspnea, or fever; chest X-ray findings; blood laboratory findings (white blood cell [WBC] counts, C-reactive protein [CRP] level, and erythrocyte sedimentation rate); and use of antibiotics [17,18].

Although a definitive diagnosis of aspiration is difficult and the diagnostic criteria for aspiration pneumonia are slightly different across studies, patients who met all of the following criteria were considered to have aspiration pneumonia in the present study: (1) the presence of both objective signs (coarse lung sounds, the presence of lung infiltration on the chest X-ray, and systemic inflammation based on blood laboratory findings, such as increased CRP levels and WBC counts) and subjective symptoms (fever, cough, and increased purulent sputum); (2) clinical suspicion of aspiration (delayed swallowing or coughing during swallowing); and (3) no evidence of microorganisms, such as *Legionella* or *Mycoplasma*, which are common pathogens in atypical pneumonia [17,18]. In addition, the clinical reports from the Internal Medicine Department were used to diagnose aspiration pneumonia.

### 2.4. The VFSS Protocol

The VFSS was performed with a fluoroscopic device and recorded as a video file. During the VFSS, patients consecutively swallowed the following materials that had a stepwise consistency: water, nectar (51–350 cP), rice porridge (351–1750 cP), and boiled rice (>1750 cP) [19]. The materials were mixed with liquid barium, and the patient swallowed them while in a relaxed sitting position. Dynamic fluoroscopic images were obtained in the anterior–posterior and lateral views and were recorded at 30 frames per second. The VFSS images were analyzed according to the Penetration–Aspiration Scale (PAS) and considered positive for aspiration if the PAS score was >5 [20].

All studies were reviewed by two physiatrists who had at least 7 years of experience in interpreting VFSS results. Patient information, including age, sex, and underlying diseases, was withheld from the interpreters. The interpreters only observed the patients using the movie files on the laptop, described their findings, and chose a feeding method (non-oral feeding versus oral feeding) based on the VFSS results.

### 2.5. Modification of the VDS

The mVDS was developed based on a study regarding the inter-rater reliability of the VDS [12,13]. Among the VDS categories, the ones with a κ value < 0.2 (bolus formation, mastication, apraxia, tongue in palate contact, and pharyngeal transit time) were modified. As mentioned by previous researchers, such categories had somewhat ambiguous guidelines and three to four multiple selectable choices, which lead to low reliability [15]. Therefore, we modified the categories according to a binary scale or deleted the ambiguous categories. The mVDS was drafted as follows (Table 1). The bolus formation and tongue to palate contact categories, which had multiple selectable choices, were deleted because of their ambiguous criteria. The lip closure and mastication categories were modified according to a binary scale of intact/not intact. The pharyngeal transit time category was based on a binary scale, but it was deleted because it had a low κ value and it was thought to have some similarity with the triggering of the pharyngeal reflex. The laryngeal elevation category had an ambiguous guideline, and the κ value was low (0.202). We changed the category to laryngeal inversion, which was reported to be an important factor in the swallowing process in a previous study because laryngeal elevation and epiglottis inversion are a result of a combination of contraction/relaxation of the suprahyoid and infrahyoid muscles [21].

Originally, to measure these VFSS findings as objective quantitative scores, the VDS with a sum of 100 points was created according to the odds ratios of various prognostic factors [12]. After modification of the parameters of the VDS, we re-balanced each category’s score of the mVDS, which had a sum of 100 points (Table 1).

### 2.6. Statistical Analysis

The intra-class correlation coefficient (ICC) model 2.1 of the VDS was used to test the inter-rater reliability based on the mVDS scores provided by the interpreters. The ICC model was used because it can be utilized for scale and ordinal variables. Ordinal variables equivalent to the weighted κ/ICC values >0.80 were considered very good, and those with κ/ICC values between 0.60 and 0.80 were considered good.

To evaluate the correlation between the mVDS and the selected feeding method and between the mVDS and the presence of aspiration pneumonia after stroke, a univariate logistic regression analysis with the enter method was used. To evaluate the accuracy of predictive factors for oral feeding or non-oral feeding based on the VFSS findings, we performed a receiver operating characteristic (ROC) analysis. Statistical analysis was conducted using the MedCalc program (MedCalc Software, Ostend, Belgium) and SPSS software version 22.0 (IBM Corp., Armonk, NY, USA).

## 3. Results

### 3.1. Patients’ Characteristics

Fifty-six stroke patients with dysphagia were enrolled in this study. Among them, 33 patients were male and 23 were female. Thirty-seven patients had ischemic stroke and 19 had hemorrhagic stroke. Thirty-eight patients had supratentorial stroke and 18 had infratentorial stroke. The modified Bathel index (MBI) was 29.60 ± 21.62, and the Mini-Mental State Examination (MMSE) was 15.25 ± 10.04. The patients’ demographic data are presented in Table 2.

### 3.2. Inter-Rater Reliability of the mVDS

The inter-rater reliability (Cronbach α value) of the total score of the mVDS was 0.886, which was consistent with very good inter-rater reliability.

### 3.3. Correlation between the mVDS and the Selected Feeding Method Based on the VFSS Findings

**Figure 1:**
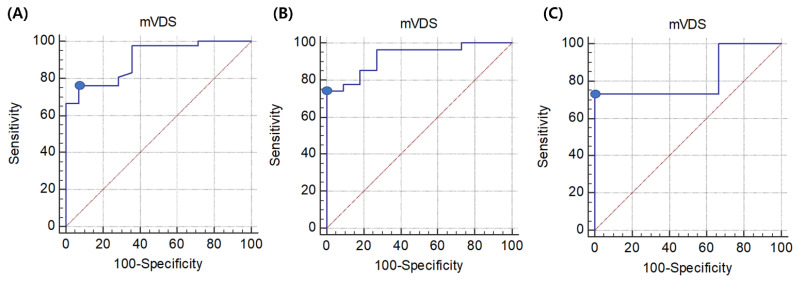
(**A**) ROC curve of the mVDS score for the selection of oral feeding in stroke patients with dysphagia. The optimal cut-off value (dots on the curves) of the mVDS score, which was obtained from the maximal Youden index, was ≤36.5 (AUC, 0.904; 95% CI, 0.795–0.966; *p* < 0.0001; sensitivity, 76.19%; specificity, 92.86%). (**B**) ROC curve of the mVDS score for the selection of oral feeding in supratentorial stroke patients with dysphagia. The optimal cut-off value obtained from the maximal Youden index was a score of ≤32 based on the mVDS (AUC, 0.926; 95% CI, 0.793–0.986; *p* < 0.0001; sensitivity, 74.07%; specificity, 100.0%) for the selection of oral feeding. (**C**) ROC curve of the mVDS score for the selection of oral feeding in infratentorial stroke patients with dysphagia. The optimal cut-off value obtained from the maximal Youden index was a score of ≤32 based on the mVDS (AUC, 0.822; 95% CI, 0.573–0.959; *p* = 0.0067; sensitivity, 73.33%; specificity, 100.0%) for the selection of oral feeding. ROC: receiver operating characteristic, AUC: area under the receiver operating characteristic curve, CI: confidence interval, mVDS: modified version of the Videofluoroscopic Dysphagia Scale.

In all patients, the mVDS score was statistically correlated with the selected feeding method (*p* < 0.05) (Table 3). In the ROC curve analysis, the area under the ROC curve (AUC) for the selected feeding method was 0.904 (95% confidence interval [CI], 0.795–0.966; *p* < 0.0001). The optimal cut-off value for the allowance of oral feeding obtained from the maximal Youden index was a score of ≤36.5 based on the mVDS (sensitivity, 76.19%; specificity, 92.86%) for the allowance of oral feeding (Figure 1A). Additionally, a score of ≤32 based on the mVDS showed a sensitivity of 66.67% and specificity of 100% for the allowance of oral feeding. For non-oral feeding, the optimal cut-off value obtained from the maximal Youden index was a score of ≥36.5 based on the mVDS (sensitivity, 92.86%; specificity, 76.19%). Additionally, a score of ≥67 showed a sensitivity of 28.57% and specificity of 100% for non-oral feeding.

In the subgroup analysis of patients with supratentorial stroke, the mVDS score was also statistically correlated with the selected feeding method (*p* < 0.05). In the ROC curve analysis, the AUC for the selected feeding method was 0.926 (95% CI, 0.793–0.986; *p* < 0.0001). The optimal cut-off value obtained from the maximal Youden index was a score of ≤32 based on the mVDS (sensitivity, 74.07%; specificity, 100.0%) for the allowance of oral feeding (Figure 1B). Additionally, a score of ≥67 showed a sensitivity of 27.27% and specificity of 100% for non-oral feeding.

In the subgroup analysis of patients with infratentorial stroke, the mVDS score was also statistically correlated with the selected feeding method (*p* < 0.05). In the ROC curve analysis, the AUC for the selected feeding method was 0.822 (95% CI, 0.573–0.959; *p* = 0.0067). The optimal cut-off value obtained from the maximal Youden index was a score of ≤34.5 based on the mVDS (sensitivity, 73.33%; specificity, 100.0%) for the allowance of oral feeding (Figure 1C). Additionally, a score of ≥51 showed a sensitivity of 33.33% and specificity of 100% for non-oral feeding.

### 3.4. Correlation between the mVDS and the Development of Aspiration Pneumonia

In the univariate logistic regression analysis, the mVDS score was significantly correlated with the presence of aspiration pneumonia after stroke (*p* < 0.05) (Table 4).

## 4. Discussion

In this study, the mVDS scores showed a statistically significant correlation with the selection of oral feeding in stroke patients with dysphagia. The result of the subgroup analysis, based on the lesion location, was also statistically significant. Interestingly, the analysis of all patients, and patients with supratentorial stroke, showed that an mVDS score of 32 had a specificity of 100%, whereas the analysis of patients with infratentorial lesions showed that an mVDS score of 34.5 had a specificity of 100%. Therefore, in general, an mVDS score of 32 is the reference point for selecting oral feeding, with a specificity of 100%.

For non-oral feeding in all stroke patients with dysphagia and in those with supratentorial stroke, an mVDS score of >60 had a specificity of 100%. However, in patients with infratentorial stroke, an mVDS score of ≥51 or higher had a specificity of 100%. One possible explanation for this discrepancy is the difference in the total number of patients in the two subgroups. The number of patients with infratentorial stroke was smaller than that of patients with supratentorial stroke, and this may have led to the different statistical outcome. Another possible explanation is the aspiration category of the mVDS. The score of the aspiration category of the mVDS did not change in accordance with how often the aspiration was detected during the VFSS. Patients with infratentorial stroke may have shown a higher incidence of aspiration during the VFSS than those with supratentorial stroke [22]. Therefore, they may have not been able to feed orally, but it would not have been accounted for in the mVDS score. In other words, the same mVDS score could still mean that the severity of swallowing difficulty may have been more significant in patients with infratentorial stroke than in those with supratentorial stroke.

According to the inter-rater reliability test, the mVDS score showed an ICC of 0.886, which was higher than that of the original VDS score (0.556) [15]. This may be due to the modification made to the categories that were somewhat ambiguous to score or had multiple choices [15]. Despite such a modification, the mVDS score was significantly correlated with the selection of oral feeding and development of aspiration pneumonia, which are important diagnoses of VFSS. Considering such correlations, it is possible to assume that the mVDS can sufficiently describe and analyze the VFSS results. However, nine of the 14 categories of the VDS have at least three selectable values, and the distinguishing between them is somewhat ambiguous [15]. This may lead to low inter-rater reliability.

In the mVDS, similar to the VDS, a higher score indicates a greater diet limitation and more severe dysphagia. The mVDS can produce numerical data regarding swallowing function by using comprehensive VFSS findings with a relatively high inter-rater reliability. Therefore, the mVDS provides more intuitive data than conventional VFSS interpretation, which is usually focused on the presence of aspiration or penetration.

There are several limitations to our study. First, the total number of enrolled patients was relatively small. Therefore, it may be challenging to make a general conclusion. Nonetheless, the results showed consistency in all stroke patients with dysphagia, the supratentorial stroke subgroup, and the infratentorial stroke subgroup. However, further studies with a greater number of participants are needed to make a more generalized conclusion. Second, the study was limited to stroke patients with dysphagia. Considering the application of the VFSS in the broad spectrum of etiology, it is crucial to apply the mVDS to diseases other than stroke.

## 5. Conclusions

The mVDS can be a useful scale for quantifying the severity of dysphagia, and it can be a helpful tool in the clinical setting and in studies to interpret the VFSS findings in stroke patients with dysphagia. In patients with an mVDS score of ≤32, it should be considered safe to select oral feeding as the feeding method.

## Figures and Tables

**Table 1:** Modified version of the Videofluoroscopic Dysphagia Scale.

Parameters		Score
lip closure	intact/not intact	0/6
Mastication	possible/not possible	0/11.5
oral transit time	≤1.5 s/>1.5 s	0/4
triggering pharyngeal swallow (swallowing reflex)	intact/delayed	0/7
epiglottis inversion	yes/no	0/13
valleculae residue	0%/<10%/≥10%, <50%/≥50%	0/3/6/9
pyriformis residue	0%/<10%/≥10%, <50%/≥50%	0/6.5/13/19.5
pharyngeal wall coating	no/yes	0/13
aspiration	intact/penetration/aspiration	0/8.5/17
total score	-	100

**Table 2:** Characteristics of stroke patients with dysphagia in the present study.

Characteristics	Mean ± Standard Deviation (Median; 25–75%)
age (year)	70.96 ± 14.456 (77.00; 63.25–80.75)
sex (male:female)	33 (58.9%): 23 (41.1%)
duration of disease (day)	422.64 ± 714.519 (255.00; 169–296.25)
PAS grade	3.80 ± 2.331 (3.00; 2.00–5.75)
MMSE score	15.2453 ± 10.03629 (17.0000; 4.5000–24.0000)
MBI score	29.6038 ± 21.61938 (26.0000; 12.0000–43.0000)
supra/infra-tentorial stroke	38 (67.9%): 18 (32.1%)
mVDS scores	
lip closure	0.32 ± 1.363 (0.00; 0.00–0.00)
massification	4.107 ± 5.5602 (0.000; 25–75%)
oral transit time	0.57 ± 1.412 (0.00; 0.00–0.00)
triggering pharyngeal swallowing	6.88 ± 0.935 (7.00; 7.00–7.00)
epiglottis inversion	0.46 ± 2.434 (0.00; 0.00–0.00)
valleculae residue	3.48 ± 1.695 (3.00; 3.00–3.00)
pyriformis residue	4.063 ± 4.5647 (3.250; 0.000–6.500)
pharyngeal wall coating	2.55 ± 5.212 (0.00; 0.00–0.00)
Aspiration	9.714 ± 6.5698 (8.500; 8.500–17.000)
total score	36.277 ± 18.6411 (32.500; 21.500–48.375)

PAS: penetration–aspiration scale, mVDS: modified Videofluoroscopic Dysphaga Scale, MMSE: Mini-Mental State Examination, MBI; modified Bathel index.

**Table 3:** Univariate logistic regression analysis (with the enter method) of the association between the modified version of the Videofluoroscopic Dysphagia Scale scores and the selection of the oral feeding method.

Patients Group	Parameter	Beta Coefficient	Standard Error	OR (95% CI)	*p*-Value
Total stroke patients with dysphagia	mVDS score	−0.114	0.031	0.892 (0.839–0.949)	<0.001
Patients with supratentorial stroke	mVDS score	−0.121	0.038	0.886 (0.823–0.954)	0.001
Patients with infratentorial stroke	mVDS score	−0.087	0.055	0.917 (0.824–1.020)	0.011

mVDS: modified version of the Videofluoroscopic Dysphagia Scale, OR: odds ratio, CI: confidence interval.

**Table 4:** Univariate logistic regression analysis (with the enter method) of the association between the modified version of the Videofluoroscopic Dysphagia Scale and the development of aspiration pneumonia.

	Parameter	Beta Coefficient	Standard Error	OR (95% CI)	*p*-Value
Development of aspiration pneumonia	mVDS score	0.051	0.020	1.053 (1.012–1.095)	<0.001

mVDS: modified version of the Videofluoroscopic Dysphagia Scale, OR: odds ratio, CI: confidence interval.

## Data Availability

The data presented in this study are available on request from the corresponding author. The data are not publicly available due to privacy reason.

## References

[1] Karkos P.D., Papouliakos S., Karkos C.D., Theochari E.G. (2009). Current evaluation of the dysphagic patient. Hippokratia.

[2] Chang M.C., Park J.-S., Lee B.J., Park D. (2021). Effectiveness of pharmacologic treatment for dysphagia in Parkinson’s disease: A narrative review. Neurol. Sci..

[3] Park D., Suh J.H., Kim H., Ryu J.S. (2019). The Effect of Four-Channel Neuromuscular Electrical Stimulation on Swallowing Kinematics and Pressures: A Pilot Study. Am. J. Phys. Med. Rehabil..

[4] Hwang J.-M., Jung H., Kim C.-H., Lee Y.-S., Lee M., Hwang S., Kim A.-R., Park D. (2021). Submandibular Push Exercise Using Visual Feedback from a Pressure Sensor in Patients with Swallowing Difficulties: A Pilot Study. Health.

[5] Park S., Cho J.Y., Lee B.J., Hwang J.-M., Lee M., Hwang S.Y., Kim K., Lee K.H., Park D. (2020). Effect of the submandibular push exercise using visual feedback from pressure sensor: An electromyography study. Sci. Rep..

[6] Chang M.C., Park S., Cho J.Y., Lee B.J., Hwang J.-M., Kim K., Park D. (2021). Comparison of three different types of exercises for selective contractions of supra- and infrahyoid muscles. Sci. Rep..

[7] Costa M.M.B. (2010). Videofluoroscopy: The gold standard exam for studying swallowing and its dysfunction. Arq. Gastroenterol..

[8] Lee J.T., Park E., Hwang J.-M., Jung T.-D., Park D. (2020). Machine learning analysis to automatically measure response time of pharyngeal swallowing reflex in videofluoroscopic swallowing study. Sci. Rep..

[9] Park D., Oh Y., Ryu J.S. (2016). Findings of Abnormal Videofluoroscopic Swallowing Study Identified by High-Resolution Manometry Parameters. Arch. Phys. Med. Rehabil..

[10] Schooling T.L. (2003). Lessons from the National Outcomes Measurement System (NOMS). Semin. Speech Lang..

[11] Lee J.H., Lee K.W., Kim S.B., Lee S.J., Chun S.M., Jung S.M. (2016). The Functional Dysphagia Scale Is a Useful Tool for Predicting Aspiration Pneumonia in Patients with Parkinson Disease. Ann. Rehabil. Med..

[12] Kim J., Oh B.-M., Kim J.Y., Lee G.J., Lee S.A., Han T.R. (2014). Validation of the Videofluoroscopic Dysphagia Scale in Various Etiologies. Dysphagia.

[13] Mo S.J., Jeong H.J., Han Y.H., Hwang K., Choi J.K. (2018). Association of Brain Lesions and Videofluoroscopic Dysphagia Scale Parameters on Patients with Acute Cerebral Infarctions. Ann. Rehabil. Med..

[14] Han T.R., Paik N.-J., Park J.W. (2001). Quantifying swallowing function after stroke: A functional dysphagia scale based on videofluoroscopic studies. Arch. Phys. Med. Rehabil..

[15] Kim D.H., Choi K.H., Kim H.M., Koo J.H., Kim B.R., Kim T.W., Ryu J.S., Im S., Choi I.S., Pyun S.B. (2012). Inter-rater Reliability of Videofluoroscopic Dysphagia Scale. Ann. Rehabil. Med..

[16] Soto-Cámara R., González-Bernal J.J., González-Santos J., Aguilar-Parra J.M., Trigueros R., López-Liria R. (2020). Age-Related Risk Factors at the First Stroke Event. J. Clin. Med..

[17] Kim G.E., Sung I.Y., Ko E.J., Choi K.H., Kim J.S. (2018). Comparison of Videofluoroscopic Swallowing Study and Radionuclide Salivagram for Aspiration Pneumonia in Children with Swallowing Difficulty. Ann. Rehabil. Med..

[18] Yu K.J., Moon H., Park D. (2018). Different clinical predictors of aspiration pneumonia in dysphagic stroke patients related to stroke lesion: A STROBE-complaint retrospective study. Medicine.

[19] Yu K.J., Park D. (2019). Clinical characteristics of dysphagic stroke patients with salivary aspiration: A STROBE-compliant retrospective study. Medicine.

[20] Borders J.C., Brates D. (2020). Use of the Penetration-Aspiration Scale in Dysphagia Research: A Systematic Review. Dysphagia.

[21] Duarte A., De Almeida J.L., Martins Ú., Magro C., Lima C., Araújo S., Pereira N., Coutinho M., Marques H. (2018). Epiglottic kinematics alterations and risk of laryngeal penetration-aspiration. Ann. Phys. Rehabil. Med..

[22] Kim Y.K., Cha J.H., Lee K.Y. (2019). Comparison of Dysphagia Between Infratentorial and Supratentorial Stroke Patients. Ann. Rehabil. Med..

